# Transcriptional Divergence of Conserved Starch Metabolism Genes During Grain Filling in *Indica* and *Japonica* Rice

**DOI:** 10.3390/cimb48050436

**Published:** 2026-04-22

**Authors:** Me-Sun Kim, Jin-Young Kim, Donghwan Shim, Kwon-Kyoo Kang, Yong-Gu Cho

**Affiliations:** 1 Institute of Agricultural Science & Technology, Chungbuk National University, Cheongju 28644, Republic of Korea; kimms0121@chungbuk.ac.kr; 2 Division of Horticultural Biotechnology, Hankyong National University, Anseong 17579, Republic of Korea; zino@hknu.ac.kr; 3 Department of Biological Sciences, Chungnam National University, Daejeon 34134, Republic of Korea; dshim104@cnu.ac.kr

**Keywords:** *Oryza sativa*, *indica*, *japonica*, starch metabolism, grain filling, gene expression, qRT-PCR, comparative analysis

## Abstract

Rice grain quality is strongly influenced by starch composition and structure, which differ between the two major cultivated *Oryza sativa* subspecies, indica and japonica. Although allelic variation in several key genes has been linked to these differences, it remains unclear whether subspecies divergence in starch metabolism is more strongly reflected in gene repertoire, structural organization, promoter composition, or transcriptional regulation. Here, we identified 52 starch metabolism-related genes representing 26 orthologous gene pairs in indica and japonica rice and compared their gene structures, predicted promoter cis-regulatory elements, and grain-filling expression patterns. The analyzed gene set was largely conserved between the two subspecies, with limited structural variation among orthologs. Although promoter analysis revealed differences in predicted cis-regulatory element composition, the strongest divergence was observed at the transcriptional level during grain filling. At 10 days after flowering (DAFs), RNA-seq profiling revealed relatively higher expression of several starch biosynthesis genes, including *SSI*, *SSIIa*, and *BEI*, in *japonica* than in *indica*. qRT-PCR further confirmed higher expression of *SSI*, *SSIIa*, *BEIIb*, and *GBSSI* in *japonica*, whereas *AGPS2b* was more highly expressed in *indica* during early grain filling. By 30 DAFs, expression of most tested genes had declined markedly in both subspecies. These findings indicate that divergence between indica and japonica is more clearly associated with transcriptional regulation during grain filling than with major differences in core starch metabolism gene content or structural organization.

## 1. Introduction

Rice (*Oryza sativa* L.) is one of the most important staple crops worldwide, and grain quality is a major determinant of consumer preference, market value, and industrial utilization. Among the biochemical components of rice grain, starch accounts for the largest proportion of endosperm dry weight and is the principal factor influencing cooking and eating quality. Variations in starch composition, particularly the relative proportions of amylose and amylopectin and the fine structure of amylopectin chains, strongly affect key physicochemical properties such as gelatinization behavior, grain hardness, pasting characteristics, and the texture of cooked rice [1,2,3]. Accordingly, understanding the molecular basis of starch metabolism is essential for clarifying how grain quality is established in rice. Starch biosynthesis in rice endosperm is controlled by a coordinated set of enzymes involved in ADP-glucose production, glucan chain elongation, branch formation, and debranching during grain filling. ADP-glucose pyrophosphorylase catalyzes the formation of ADP-glucose, the principal glucosyl donor for starch synthesis, whereas granule-bound starch synthase I, encoded by the *Wx* locus, plays a central role in amylose synthesis [4,5,6,7,8]. In contrast, amylopectin biosynthesis is mainly governed by soluble starch synthases, branching enzymes, and debranching enzymes, whose coordinated activities determine amylopectin architecture and starch granule properties [4,7,8]. Because starch accumulation depends on the integrated action of multiple enzymes rather than on a single gene, comparative analysis of the broader starch metabolism-related gene set may provide a more informative view of subspecies differences than analyses restricted to individual loci alone.

The two major cultivated *Oryza sativa* subspecies, *indica* and *japonica*, differ substantially in grain characteristics, starch physicochemical properties, and eating quality [4,5]. In particular, allelic variation in key genes such as *Wx/GBSSI* and *SSIIa* has long been associated with differences in amylose content, amylopectin chain-length distribution, and gelatinization behavior between the two subspecies [6,9,10]. The high-amylose *Wxa* allele is predominantly distributed in *indica*, whereas the *Wxb* allele associated with reduced amylose synthesis is common in *japonica* [9]. Likewise, variation in *SSIIa* has been linked to subspecies differences in amylopectin structure and thermal properties [10]. These findings indicate that starch-related divergence between *indica* and *japonica* is associated not only with starch quantity, but also with differences in the molecular regulation of starch biosynthesis. Despite these well-characterized differences in a small number of major genes, most genes involved in starch metabolism are shared between *indica* and *japonica*. This raises an important question as to whether subspecies divergence in starch metabolism is more clearly reflected in gene repertoire, structural organization, promoter composition, or transcriptional regulation. Previous transcriptomic studies have shown that many starch biosynthetic genes display strong tissue specificity and developmental regulation in rice, particularly during grain filling [11,12,13,14]. Moreover, functional studies of individual enzymes have demonstrated that genes such as *SSI*, *SSIIa*, *BEIIb*, and *GBSSI* make distinct contributions to amylopectin and amylose biosynthesis and thereby influence starch physicochemical properties [7,10,15,16]. These observations suggest that comparative analysis of starch metabolism-related genes at multiple levels may provide useful insight into how a conserved biosynthetic pathway is differentially regulated in *indica* and *japonica*. However, comparative transcriptomic studies specifically addressing starch metabolism-related genes between *indica* and *japonica* remain limited, particularly those integrating orthologous relationships, structural conservation, predicted promoter composition, and grain-filling expression patterns within a single comparative framework. In particular, it remains unclear which level of variation most clearly distinguishes *indica* and *japonica* within a conserved starch metabolism-related gene set.

Therefore, in this study, we performed a comparative analysis of starch metabolism-related genes in *indica* and *japonica* rice. We identified orthologous gene pairs associated with major starch metabolic processes and examined their structural features, conserved motifs, and predicted cis-regulatory elements. We further analyzed their expression patterns across tissues and grain-filling stages using transcriptome data and validated representative genes by qRT-PCR. Through this approach, we aimed to determine whether differences between *indica* and *japonica* are more clearly reflected in gene repertoire, structural organization, promoter composition, or transcriptional behavior during grain filling.

## 2. Materials and Methods

### 2.1. Identification of Starch Metabolism-Related Genes in Oryza sativa

Genome sequence and annotation data for *Oryza sativa* subsp. *japonica* cv. Nipponbare and *O. sativa* subsp. *indica* cv. Shuhui498 (R498) were obtained from RAP-DB and MBKbase, respectively (accessed on 1 April 2026). Starch metabolism-related genes were identified based on functional annotation and sequence homology to previously reported rice starch biosynthesis- and starch metabolism-associated genes. The identified genes were classified into major functional groups, including ADP-glucose pyrophosphorylase (AGPase), soluble starch synthases (SSs), granule-bound starch synthases (GBSSs), branching enzymes (BEs), debranching enzymes (DBEs), starch phosphorylase-related proteins, and glucose-6-phosphate transporter 1 (*OsGPT1*). Orthologous gene pairs between the two subspecies were assigned from a defined set of previously characterized rice starch metabolism-related genes based on conserved functional annotation, locus correspondence in the reference genome annotations, and the closest sequence homolog relationship between the two genomes. Because the aim of this study was to comparatively characterize known starch metabolism-related genes in indica and japonica rice, ortholog assignment was performed using curated functional equivalence and annotation consistency within this targeted gene set.

### 2.2. Structural and Sequence Analyses of Starch Metabolism-Related Genes

Chromosomal positions of the identified genes were visualized using MapGene2Chromosome v2.0 (http://mg2c.iask.in/mg2c_v2.0/, accessed on 1 April 2026). Exon–intron structures were analyzed using the Gene Structure Display Server (GSDS) by comparing full-length coding sequences with their corresponding genomic DNA sequences (GSDS; http://gsds.gao-lab.org/ accessed on 1 April 2026). Conserved protein motifs were identified using MEME Suite, with the maximum number of motifs set to 10 and all other parameters kept at their default settings (https://meme-suite.org/meme/ accessed on 1 April 2026). The relative positions of the predicted motifs in each protein were visualized based on the MEME output, and the consensus motif sequences are summarized in Supplementary Table S1. Sequence logos representing motif conservation were also generated from the MEME results (Supplementary Figure S1).

### 2.3. Cis-Acting Regulatory Element Analysis

Promoter regions were defined as the 3000 bp upstream sequences from the translation start site of each starch metabolism-related gene. Promoter sequences were retrieved from RAP-DB (https://rapdb.dna.affrc.go.jp/, accessed on 1 April 2026). Cis-acting regulatory elements within these promoter regions were identified and annotated using PlantCARE (http://bioinformatics.psb.ugent.be/webtools/plantcare/html, accessed on 1 April 2026). Predicted cis-elements were subsequently classified according to their annotated regulatory functions, including light responsiveness, phytohormone signaling, growth and development, and stress responsiveness.

### 2.4. Transcriptome Analysis During Grain Filling

Temporal and spatial expression profiles of starch metabolism-related genes were analyzed using publicly available normalized transcriptome data obtained from RiceXPro v3.0 (http://ricexpro.dna.affrc.go.jp/, accessed on 1 April 2026). Expression profiles across tissues and developmental stages were examined to compare relative transcriptional patterns between indica and japonica, with particular emphasis on grain-filling stages. Among the identified starch metabolism-related genes, 24 orthologous gene pairs with available and comparable expression profiles were selected for analysis. Heatmaps were generated from normalized expression values provided by the database and subsequently transformed to row Z-scores to enable comparison of relative expression patterns across samples. Genes were clustered based on expression similarity. The transcriptome dataset was used for exploratory comparison of relative transcript accumulation patterns across tissues, developmental stages, and subspecies.

### 2.5. Plant Materials and RNA Preparation for qRT-PCR

Rice plants representing the indica and japonica subspecies were grown in a greenhouse under a 14 h light/10 h dark photoperiod, with temperatures maintained at 28–30 °C during the day and 22–24 °C at night. Developing grains were harvested at 10, 20, and 30 days after flowering (DAFs) to represent key stages of grain filling. Grain tissues were immediately frozen in liquid nitrogen and stored at −80 °C until use. Total RNA was extracted using the RNeasy Plant Mini Kit (QIAGEN, Hilden, Germany) according to the manufacturer’s instructions. RNA concentration and purity were assessed using a NanoDrop One spectrophotometer (Thermo Fisher Scientific, Waltham, MA, USA). Only RNA samples with acceptable purity were used for subsequent analyses.

### 2.6. qRT-PCR Analysis

First-strand cDNA was synthesized from total RNA using Oligo(dT) primers and ReverTra Ace™ qPCR RT Master Mix (TOYOBO, Osaka, Japan). qRT-PCR was performed using iQ™ SYBR Green Supermix (Bio-Rad, Hercules, CA, USA) on a CFX96 Real-Time PCR Detection System (Bio-Rad Laboratories, Hercules, CA, USA), following the manufacturers’ instructions. Gene-specific primers were used for representative starch metabolism-related genes, including *OsAGPL1*, *OsAGPS2b*, *OsSSI*, *OsSSIIa*, *OsBEIIb*, and *OsGBSSI*, and the primer sequences are listed in Supplementary Table S2. *OsACTIN* (LOC_Os03g50885.1) was used as the internal control. Relative expression levels were calculated using the 2^−ΔΔCt^ method. Three independent biological replicates were analyzed for each sample. Differences in gene expression between indica and japonica at each sampling stage were evaluated using Student’s *t*-test for pairwise comparison. Because the qRT-PCR analysis was designed as targeted validation of expression patterns in two groups at each time point, the statistical analysis was used to support comparative interpretation of the observed trends.

## 3. Results

### 3.1. Identification of Orthologous Genes in Indica and Japonica Rice

**Figure 1:**
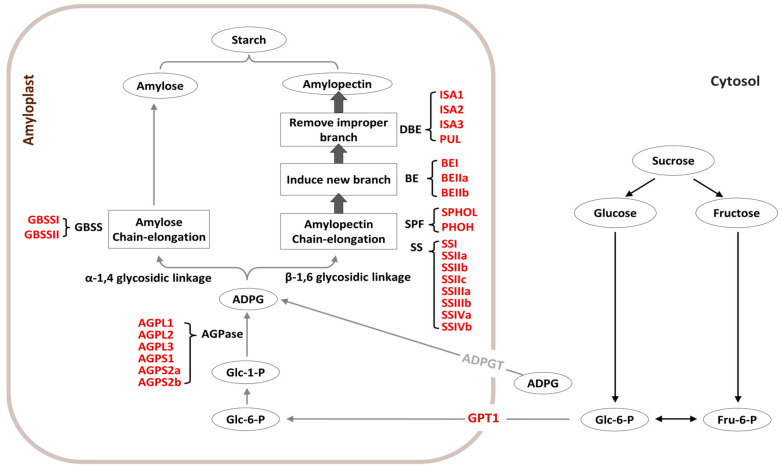
Schematic overview of starch metabolism in rice endosperm and the major gene groups analyzed in this study. Circles indicate major metabolites, and rectangles indicate the principal enzymatic steps involved in ADP-glucose production, amylose synthesis, amylopectin elongation, branching, and debranching. The starch metabolism-related genes analyzed in this study are grouped according to their functional classes, including AGPase, SS, GBSS, BE, DBE, starch phosphorylase family members, and GPT1.

To examine whether differences in starch-related traits between *indica* and *japonica* are associated with differences in major starch metabolism-related genes, we first identified orthologous genes in the two subspecies. A total of 52 starch metabolism-related genes, representing 26 orthologous gene pairs, were identified in the two major *Oryza sativa* subspecies (Table 1; Supplementary Tables S3 and S4). These genes were assigned to the major functional groups associated with rice starch metabolism, including AGPase, SSs, GBSSs, BEs, DBEs, starch phosphorylase family members, and GPT1. Together, these orthologous pairs covered the principal enzymatic steps of starch metabolism in rice endosperm, including ADP-glucose production, amylose synthesis, amylopectin elongation, branch formation, and debranching (Figure 1). Importantly, each gene identified in one subspecies had a clear orthologous counterpart in the other, and no lineage-specific gene gain or loss was detected within the analyzed gene set. The corresponding gene family sizes were also identical between the two genomes. These results indicate that the major starch metabolism-related genes are conserved between *indica* and *japonica* and that differences in starch-related traits are unlikely to be explained simply by the presence or absence of core pathway genes.

### 3.2. Structural Comparison of Orthologous Genes in Indica and Japonica

**Figure 2:**
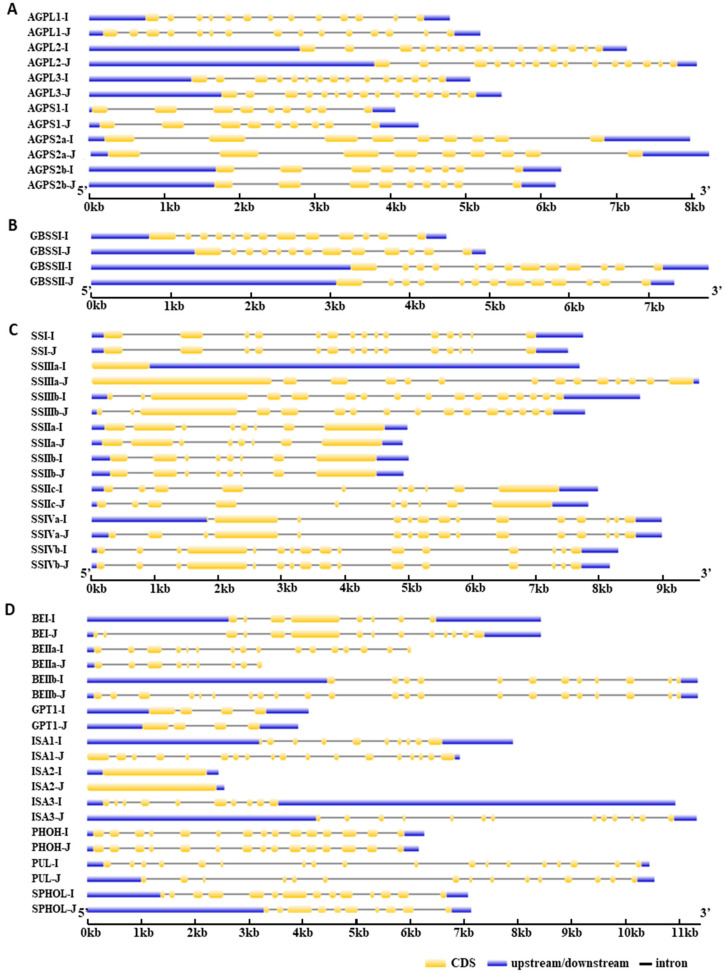
Exon–intron structures of starch metabolism-related genes in the two *Oryza sativa* subspecies, *indica* and *japonica*. Gene structures were visualized using GSDS by aligning coding sequences with their corresponding genomic sequences. Yellow boxes indicate coding sequences (CDSs), blue boxes indicate untranslated regions (UTRs), and black lines indicate introns. (**A**) ADP-glucose pyrophosphorylase (AGPase) genes. (**B**) Granule-bound starch synthase (GBSS) genes. (**C**) Soluble starch synthase (SS) genes. (**D**) Starch branching and debranching enzyme genes.

**Figure 3:**
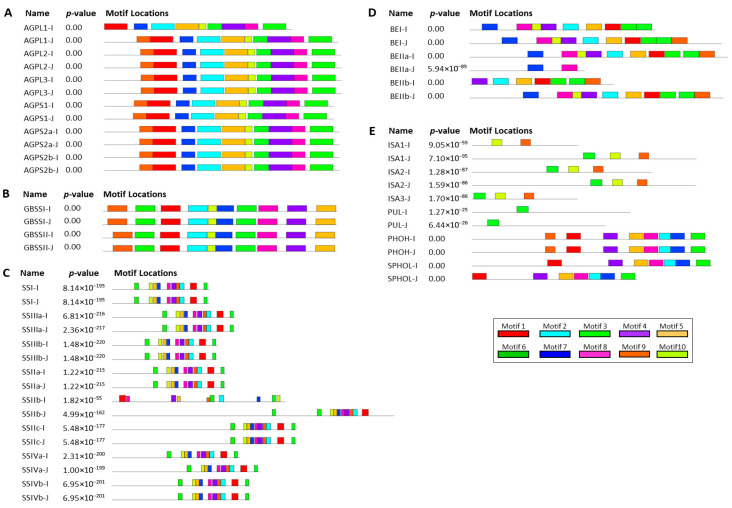
Distribution of conserved motifs in starch metabolism-related proteins from *indica* and *japonica* rice. Conserved motifs were identified using MEME, and each colored box represents a distinct motif. The relative positions and arrangement of motifs within each protein are shown. (**A**) ADP-glucose pyrophosphorylase-related proteins. (**B**) Granule-bound starch synthase-related proteins. (**C**) Soluble starch synthase-related proteins. (**D**) Starch branching enzyme-related proteins. (**E**) Starch debranching enzyme-related proteins.

To examine whether structural variation in starch metabolism-related genes distinguishes *indica* and *japonica*, we compared the phylogenetic relationships, chromosomal distribution, exon–intron organization, and conserved motif composition of orthologous genes in the two subspecies (Figure 2 and Figure 3; Supplementary Figures S2 and S3; Supplementary Table S1). Most orthologous genes were mapped to corresponding chromosomal positions in *indica* and *japonica*, and no apparent gain-or-loss pattern was detected at the genomic distribution level (Supplementary Figure S3). Phylogenetic analysis further showed that most proteins clustered according to their annotated functional classes in both separate and combined trees (Supplementary Figure S2). Comparison of exon–intron organization showed that gene structures were largely similar between orthologous pairs (Figure 2). This pattern was most evident in AGPase- and GBSS-related genes. Within the soluble starch synthase family, *SSI*, *SSIIa*, and *SSIV*-related genes also showed similar exon organization, whereas *SSIIIa* displayed clearer differences in exon number and exon length between *indica* and *japonica*. Branching and debranching enzyme-related genes showed a similar trend: most orthologs were structurally comparable, whereas selected genes, including *BEIIa*, *PUL*, and several *ISA*-related genes, showed moderate differences in exon arrangement. A comparable pattern was observed at the protein level (Figure 3; Supplementary Table S1). Proteins within the same functional groups generally shared similar motif composition and motif order in the two subspecies, although limited motif variation was detected in a small number of proteins, most clearly in *SSIIIa* and several branching and debranching enzyme-related members. These results indicate that the overall structural organization of starch metabolism-related genes is largely conserved between *indica* and *japonica*, with detectable variation restricted to a limited subset of genes.

### 3.3. Comparison of Predicted Cis-Regulatory Elements in Promoter Regions

**Figure 4:**
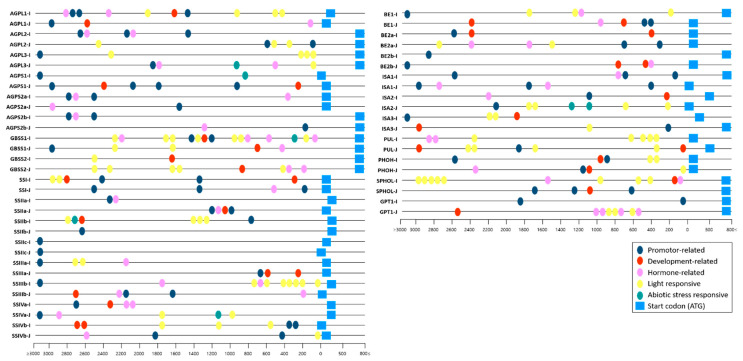
Distribution of predicted cis-acting regulatory elements in the promoter regions of starch metabolism-related genes in indica and japonica rice. Promoter sequences 3000 bp upstream of each gene were analyzed using PlantCARE, and the locations of predicted cis-acting elements are shown for individual genes.

**Figure 5:**
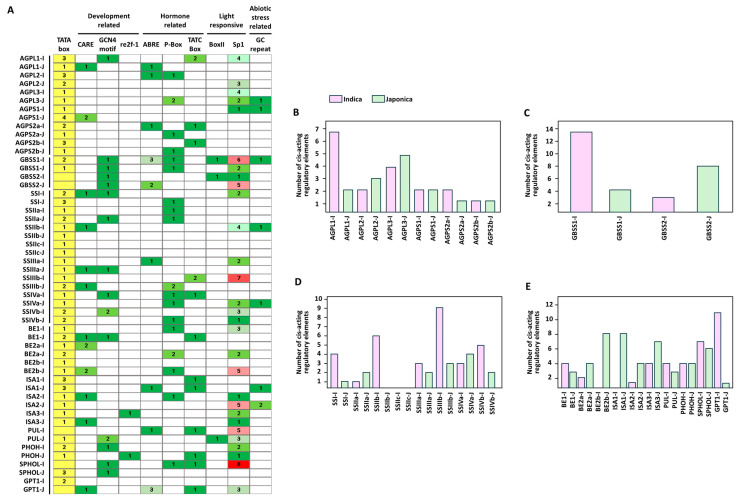
Analysis of cis-acting regulatory elements in the promoter regions of starch metabolism-related genes in *indica* and *japonica* rice. Promoter sequences 3000 bp upstream of the translation start site were analyzed using PlantCARE. (**A**) Functional classification of predicted cis-acting elements identified in starch metabolism-related gene promoters. (**B**–**E**) Total numbers of predicted cis-acting elements in the promoter regions of starch metabolism-related genes from *indica* and *japonica*, grouped according to functional category.

To examine potential regulatory divergence between *indica* and *japonica*, we analyzed predicted cis-regulatory elements within the 3 kb upstream promoter regions of starch metabolism-related genes (Figure 4 and Figure 5). In both subspecies, most promoters contained multiple putative cis-elements associated with light responsiveness, phytohormone signaling, growth and development, and stress responses, indicating that these genes are likely subject to complex transcriptional regulation. Comparison of element distribution patterns showed that the overall promoter architectures were broadly conserved between the two subspecies, although differences in the relative abundance of specific cis-element categories were observed (Figure 4 and Figure 5A). Notably, light-responsive elements were more prevalent in *indica* promoters, whereas *japonica* promoters showed relatively higher proportions of growth- and development-related as well as hormone-responsive elements. When genes were further grouped by enzyme class, *indica* promoters tended to harbor larger numbers of predicted cis-elements in AGPase, GBSS, and soluble starch synthase-related genes, whereas *japonica* promoters showed relatively greater cis-element abundance in branching enzyme- and debranching enzyme-related genes (Figure 5B–E). Together, these results indicate that, despite the overall conservation of promoter architecture in orthologous starch metabolism-related genes, subspecies-specific differences in predicted cis-regulatory element composition are present between indica and japonica.

### 3.4. Expression Profiles During Grain Filling in Indica and Japonica

**Figure 6:**
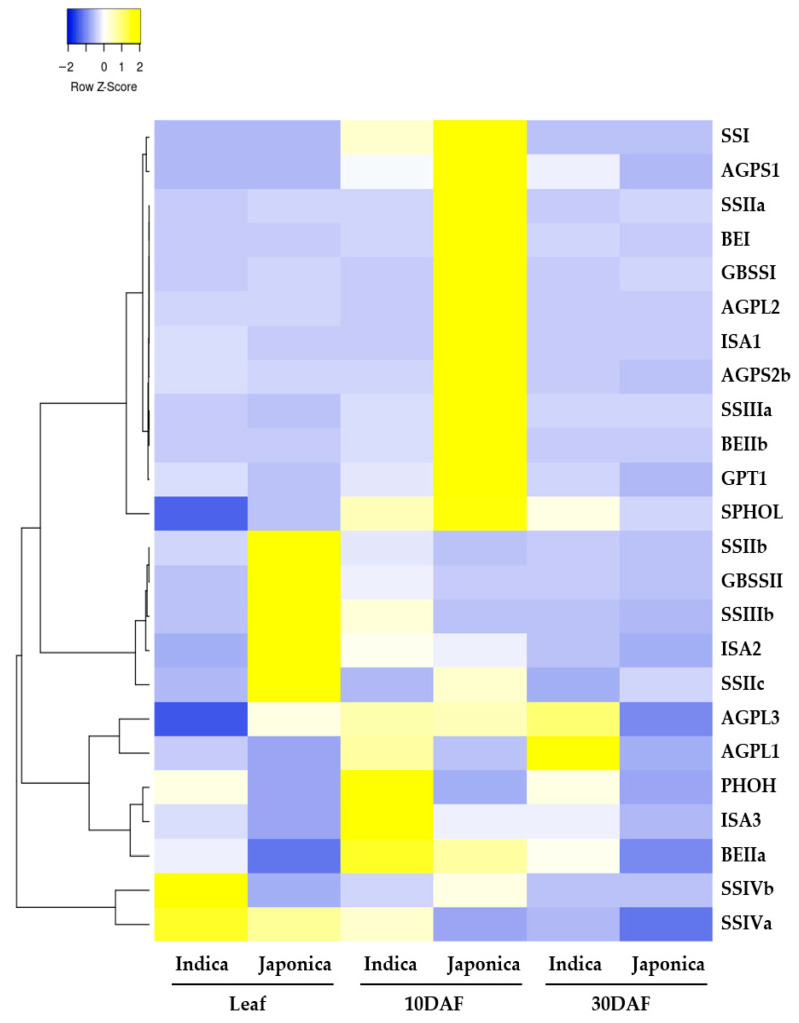
Transcriptomic expression profiles of 24 orthologous starch metabolism-related gene pairs during grain filling in indica and japonica rice. The heatmap shows relative expression patterns across tissues and developmental stages based on normalized expression values obtained from RiceXPro. For visualization, expression values for each gene were transformed to row-wise Z-scores, so that color intensity reflects relative transcript accumulation within each gene across samples. Genes were clustered according to expression similarity.

To explore transcriptional patterns during grain filling, transcriptome expression profiles were compared across tissues and developmental stages in *indica* and *japonica* using publicly available transcriptome data (Figure 6). Hierarchical clustering of normalized transcript levels revealed clear tissue- and stage-dependent expression patterns among the analyzed genes. In leaf tissues, genes associated with transient starch metabolism showed subspecies-associated differences in relative transcript accumulation: *OsSSIVa* and *OsSSIVb* showed relatively higher transcript accumulation in *indica*, whereas *OsGBSSII*, together with *OsSSIIb*, *OsSSIIc*, *OsSSIIIb*, and *OsISA2*, showed relatively higher transcript accumulation in *japonica*. During early grain filling (10 DAFs), several core endosperm starch biosynthesis genes, including *OsSSI*, *OsSSIIa*, and *OsBEI*, showed relatively higher transcript accumulation in *japonica* than in *indica*. In contrast, *OsAGPL1* and *OsPHOH* showed relatively higher transcript accumulation in *indica* at the same stage. By 30 DAFs, transcript levels of most genes had declined markedly in both subspecies and approached basal levels. Overall, these transcriptome profiles provide an exploratory view of subspecies-associated expression patterns during grain filling.

### 3.5. qRT-PCR Validation of Grain-Filling Expression Patterns

**Figure 7:**
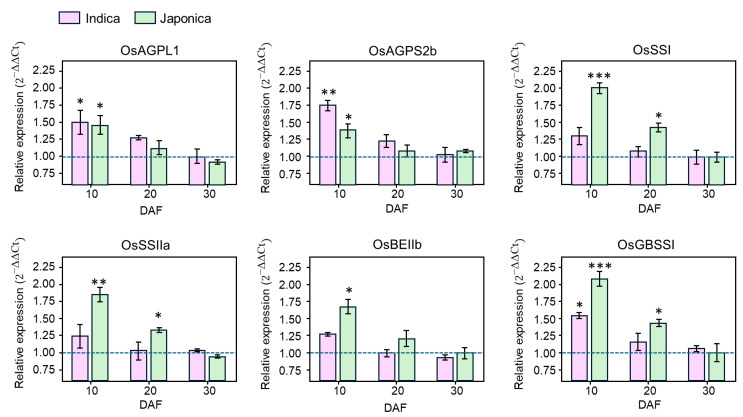
qRT-PCR validation of representative starch metabolism-related genes during grain filling in *indica* and *japonica* rice. Quantitative real-time PCR was performed for representative genes involved in ADP-glucose production (*OsAGPL1* and *OsAGPS2b*), glucan chain elongation (*OsSSI* and *OsSSIIa*), branching (*OsBEIIb*), and amylose synthesis (*OsGBSSI*). Relative expression levels were calculated using the 2^−ΔΔCt^ method with *OsActin* as the internal control. Bars represent mean ± SD of three biological replicates. Asterisks indicate significant differences between *indica* and *japonica* (* *p* < 0.05, ** *p* < 0.01, *** *p* < 0.001).

To validate the expression patterns observed in the RNA-seq profiles during grain filling, qRT-PCR was performed for representative genes involved in major steps of starch biosynthesis (Figure 7). The selected genes included *OsAGPL1* and *OsAGPS2b*, associated with ADP-glucose production, *OsSSI* and *OsSSIIa*, involved in glucan chain elongation, *OsBEIIb*, associated with branch formation, and *OsGBSSI*, which plays a central role in amylose synthesis. Overall, the qRT-PCR results were generally consistent with the RNA-seq expression patterns and supported subspecies-biased expression during early grain filling. At 10 DAFs, *SSI* and *SSIIa* showed higher expression in japonica than in indica, with relative expression values of approximately 2.0 vs. 1.3 and 1.8 vs. 1.2, respectively. *BEIIb* and *GBSSI* also showed higher expression in *japonica*, with values of approximately 1.7 vs. 1.3 and 2.1 vs. 1.5, respectively. By contrast, *AGPS2b* showed higher expression in *indica* than in *japonica* at the same stage, with values of approximately 1.7 and 1.4, respectively, whereas *AGPL1* showed only a modest subspecies difference. As grain filling progressed, expression levels of most tested genes declined toward 30 DAFs in both subspecies, approaching basal levels. Taken together, these results support the view that transcriptional divergence between *indica* and *japonica* is most evident during early grain filling.

## 4. Discussion

Rice grain quality is strongly influenced by starch composition and structure, particularly the relative proportions of amylose and amylopectin and the fine structure of amylopectin chains [15,16,17]. In the present study, we compared orthologous starch metabolism-related genes between *indica* and *japonica* at the levels of gene identity, structural organization, promoter composition, and grain-filling expression. Integrating these layers of evidence, our results indicate that the clearest distinction between the two subspecies lies not in major differences in gene repertoire, but in the developmental regulation of conserved starch metabolism-related genes during grain filling (Table 1; Figure 1, Figure 2, Figure 3, Figure 4, Figure 5, Figure 6 and Figure 7; Supplementary Figures S1–S3; Supplementary Tables S1–S4). This conclusion is consistent with current views that grain-filling behavior and endosperm starch properties are shaped by coordinated regulatory control of shared biosynthetic components rather than by simple gain or loss of pathway genes [11].

Our first major inference is that the core starch metabolism-related framework is broadly conserved between *indica* and *japonica*. The identification of 26 orthologous gene pairs spanning the principal enzymatic steps of endosperm starch metabolism, together with the absence of lineage-specific gain or loss within the analyzed gene set, argues against large-scale pathway differentiation at the gene-content level (Table 1; Figure 1). This conclusion is further supported by the largely similar exon–intron organizations and conserved motif compositions observed for most orthologous pairs (Figure 2 and Figure 3; Supplementary Figures S2 and S3; Supplementary Table S1). Although a subset of genes, including *SSIIIa*, *BEIIa*, *PUL*, and several *ISA*-related members, showed detectable structural variation, these differences were limited relative to the overall conservation pattern. Thus, the present data support the interpretation that subspecies divergence in starch-related traits is unlikely to be explained primarily by broad restructuring of the core starch metabolic pathway.

A second inference is that regulatory divergence is more plausibly reflected in promoter composition and expression behavior than in gene presence or gross structure. Promoter analysis revealed differences in the relative abundance of predicted cis-regulatory categories between the two subspecies, with light-responsive elements more frequently represented in *indica* and growth/development- and hormone-related elements more prominent in *japonica* (Figure 4 and Figure 5). These differences suggest that orthologous starch metabolism-related genes may be embedded in partially distinct regulatory contexts. However, the cis-element analysis in the present study was based on in silico prediction alone and should therefore be interpreted cautiously. Predicted promoter composition can indicate possible regulatory divergence, but it does not by itself demonstrate that specific cis-elements are functionally responsible for the observed expression outcomes [18,19]. Accordingly, the promoter comparisons reported here are best regarded as a framework for generating regulatory hypotheses rather than as direct evidence of causal promoter function. This interpretation is in line with recent work emphasizing that mechanistic inference about cis-regulatory control requires functional assays such as promoter–reporter analyses, transcription factor binding tests, or chromatin-level measurements.

The strongest support for subspecies divergence in the present dataset emerged from the expression layer. RNA-seq profiling revealed clear tissue- and developmental stage-dependent differences in transcript accumulation, with the most evident contrast observed during early grain filling at 10 DAFs (Figure 6). This trend was further supported by qRT-PCR analysis of representative genes involved in ADP-glucose production, glucan chain elongation, branch formation, and amylose synthesis (Figure 7; Supplementary Table S2). Considered together, these results suggest that *indica* and *japonica* differ most clearly in the temporal deployment of conserved starch metabolism-related genes during a critical phase of endosperm development. This interpretation is biologically plausible because early grain filling corresponds to an active stage of starch deposition, when coordinated regulation of AGPase, starch synthases, branching enzymes, and associated factors is expected to influence final starch architecture and grain-quality traits [11,20,21,22,23,24]. The known functional importance of *GBSSI*, *SSIIa*, *SSI*, and *BEIIb* for amylose synthesis, amylopectin chain-length distribution, and starch physicochemical properties further supports the idea that developmental differences in transcript accumulation may contribute to subspecies-specific starch behavior [6,8,15,17,22,23,24]. At the same time, the present data do not support a simple interpretation in which one subspecies uniformly exhibits stronger pathway activity than the other. Rather, the overall pattern is more consistent with differences in timing, coordination, and relative balance across multiple functional modules within a shared starch metabolic network.

Several limitations of this study should be acknowledged. First, the comparative framework was defined at the orthologous gene level and therefore did not resolve allele-level or allele-specific transcriptional behavior within individual loci, an issue that is particularly relevant for genes such as *Wx* and *SSIIa* that are already known to harbor functionally important allelic variation [6,10]. Second, although the promoter analysis identified differences in predicted cis-regulatory composition, direct functional validation of promoter activity and regulatory interactions was not performed. Third, the expression comparisons were based on public transcriptome profiles together with targeted qRT-PCR validation and thus do not fully capture the environmental variation that may influence grain filling and starch metabolism in different genetic backgrounds [5,11]. These limitations indicate that the present study should be viewed as establishing a comparative regulatory framework rather than a complete mechanistic model. Future studies incorporating allele-specific expression analysis, promoter–reporter assays, transcription factor binding analysis, and chromatin or epigenetic profiling will be important for testing whether the putative regulatory differences identified here are functionally linked to subspecies-specific starch metabolism [18,19,20]. Even with these limitations, the present work provides a useful synthesis of orthology, structural conservation, promoter architecture, and developmental expression, and it highlights transcriptional regulation during grain filling as a key level at which grain-quality divergence between *indica* and *japonica* may emerge.

## 5. Conclusions

In this study, we performed a comparative analysis of starch metabolism-related genes in the two major *Oryza sativa* subspecies, *indica* and *japonica*. The analyzed gene set was broadly conserved between the two subspecies at the levels of gene identity and overall structural organization, whereas more evident differences were observed in predicted promoter composition and, most prominently, in transcript accumulation during grain filling. In particular, several key starch biosynthesis genes showed higher expression in *japonica* during early grain filling, whereas a subset of genes showed relatively higher expression in *indica*. These results indicate that subspecies variation in rice grain quality is more likely to be associated with differential regulation of conserved starch metabolism genes during grain filling than with major differences in pathway composition itself.

## Figures and Tables

**Table 1:** Orthologous starch metabolism-related genes identified in *indica* and *japonica* rice. Orthologous pairs were assigned based on annotation correspondence and sequence similarity. Genes were classified according to their functional roles in rice starch metabolism.

Functional Group	Gene	*Indica* Locus ID	*Japonica* Locus ID
AGPase	*OsAGPL1*	OsR498G0307002800.01	Os03g0735000
AGPase	*OsAGPL2*	OsR498G0101598700.01	Os01g0633100
AGPase	*OsAGPL3*	OsR498G0511403400.01	Os05g0580000
AGPase	*OsAGPS1*	OsR498G0917015400.01	Os09g0298200
AGPase	*OsAGPS2a*	OsR498G0815850400.01	Os08g0345800
AGPase	*OsAGPS2b*	OsR498G0815850400.01	Os08g0345800
SS	*OsSSI*	OsR498G0611665000.01	Os06g0160700
SS	*OsSSIIa*	OsR498G0611935700.01	Os06g0229800
SS	*OsSSIIb*	OsR498G0204761600.01	Os02g0744700
SS	*OsSSIIc*	OsR498G1018786300.01	Os10g0437600
SS	*OsSSIIIa*	OsR498G0815345700.01	Os08g0191433
SS	*OsSSIIIb*	OsR498G0409312000.01	Os04g0624600
SS	*OsSSIVa*	OsR498G0101909600.01	Os01g0720600
SS	*OsSSIVb*	OsR498G0511243300.01	Os05g0533600
GBSS	*OsGBSSI*	OsR498G0611577100.01	Os06g0133000
GBSS	*OsGBSSII*	OsR498G0714096400.01	Os07g0412100
BE	*OsBEI*	OsR498G0613332800.01	Os06g0726400
BE	*OsBEIIa*	OsR498G0408518200.01	Os04g0409200
BE	*OsBEIIb*	OsR498G0203981600.01	Os02g0528200
DBE	*OsISA1*	OsR498G0816481400.01	Os08g0520900
DBE	*OsISA2*	OsR498G0510777600.01	Os05g0393700
DBE	*OsISA3*	OsR498G0917656000.01	Os09g0469400
DBE	*OsPUL*	OsR498G0407601700.01	Os04g0164900
SPH	*OsPHOH*	OsR498G0102345000.01	Os01g0851700
SPH	*OsSPHOL*	OsR498G0307078200.01	Os03g0758100
Transport	*OsGPT1*	OsR498G0815333700.01	Os08g0187800

## Data Availability

The original contributions presented in this study are included in the article/Supplementary Material. Further inquiries can be directed to the corresponding authors.

## Supplementary Materials

The following supporting information can be downloaded at https://www.mdpi.com/article/10.3390/cimb48050436/s1.
