# Supplementary material for: Transcriptional Divergence of Conserved Starch Metabolism Genes During Grain Filling in Indica and Japonica Rice
Source: Curr Issues Mol Biol. 2026 Apr 22;48(5):436. doi: 10.3390/cimb48050436 (PMC13204041; doi:10.3390/cimb48050436)
Supplement: Supplementary file 1 [file cimb-48-00436-s001.zip › cimb-4265970-supplementary.pdf]

Supplementary Table S1. Consensus motif sequences of conserved motifs identified in starch metabolism-related proteins of indica and japonica rice.

Protein Group	Motif	Consensus Sequence (Regular expression)
ADP-glucose pyrophosphorylase -related proteins	1	PAVPJGANYRLIDIPVSNCLNSGINKIYVLTQFNSASLNRHLSRAYGNNI
	2	LAGDHLYRMDYMKFIQAHRETBADITVAALPMDESRASAFGLMKIDDEGR
	3	PIGIGENTKIRNAIIDKNARIGDNVKIINVDGVQEADRETEGYYIKSGIV
	4	PPPDFSFYDRSAPIYTSPRYLPPSKVLDADITDSIIGEGCVJKNCTIEHS
	5	FAEKPKGEDLKAMMVDTTILGLDDDRAKEMPYIASMGIYVFKKBVLLNLL
	6	EMRVQAYLYDGYWEDIGTIEAFYBANLGJTE
	7	AQQSPENPNWFQGTADAVRQYLWLFEEHN
	8	VVGJRSRISEGAIJEDSLLMGADYYETEA
	9	PDASTSVLGIILGGGAGTRLYPLTKKRAK
	10	ERFPGANDFGSEIJP
Granule-bound starch synthase-related proteins	1	FHCYKRGVDRVFIDHPSFLEKVWGKTGEKJYGPDTGDDYKDNQLRFSLLC
	2	YGEDVVFVANDWHTGPLPSYLKNNYQPKGIYVNAKVAFCIHNIAYQGRFA
	3	FIGAECAPWSKTGGLGDVLGGLPPALAANGHRVMVIVPRYDQYKDAWDTN
	4	VPSRFEPCGLIQLQGMRYGVPPACASTGGLVDTVKEGKTGFHMGRFNVDC
	5	YZEMVRNCMAQDLSWKGPAKKWEEVLLGLGVAGSQPGIEGDEIAPLAKEN
	6	TGIVNGMDVYEWDPAKDKYISVKYDATTVTEAKALNKEALQAEVGLPVDR
	7	DKPVEGRKINWMKAGILEADRVLTVSPHYVEELISGPDKGVEL
	8	FIGRLEEQKGPDILAAAIPEFVZEDVQIILLGTGKKKFEELLKLLEEKYP
	9	TGFGQADRYGPRFLKRDGFQGRKPKHPAGGDATSLGICTKPRRSPKPQVS
	10	DYPLLNLPDRFRPSFDFIDGH
Soluble starch synthase-related proteins	1	HRITAGADVLLMPSRFEPCGLTQLYAMRYGTVPVVHATGGL
	2	DVPLIGFIGRLDPQKGVDLIKHAIPWIAE
	3	TGGLGDVVGALPKALARRGHRVEVVLP
	4	HDWKFNGIVNGIDTDEWNPSTDRHLKVDY
	5	IFHCNDWHTALVPVLLKAYYA
	6	GLQKRGMSQDFSWDHAASQYE
	7	MSYARSVLVIHNIAYQGRGPA
	8	DRVTTVSPGYAWEVKTEGGGW
	9	DLSGKAACKAALQKELGLQVR
	10	KRMILFCKAALEVPWQSPCGG
Starch branching enzyme-related proteins	1	WKMGDIVHTLTNRRWSEKCVTYAESHDQAJVGDKTIAFWLMDKDMYDFMA
	2	HHWMWDSRLFNYGNWEVLRFLLSNARWWLEEYKFDGFRFDGVTSMMYTHH
	3	RPATPSIDRGIALHKMIRLITMGLGGEGYLNFMGNEFGHPEWIDFPRAPQ
	4	FGYHVTNFFAPSSRFGRPEDLKSLIDKAHELGLVVLMDVVHSHASNNTLD
	5	MLVNDLIHGLYPEAITIGEDVSGMPTFCLPVQDGGVGFDYRLHMAVPDKW
	6	NWSYDKCRRRFDLGDADYLRYKGMNEFDQAMQALEEKYGFMTSDHQYISR
	7	IDQHEGGLEEFSRGYEKFGFNRSAEGITYREWAPGAQSAALVGDFNNWNP
	8	PYBGIYYDPPEEEKYVFKHPQPKRPBSLRIYESHVGMSSPEPEINTYANF
	9	HEEDKVIIFEKGDLVFVFNFHWSNSYFDYRVGCLKPGKYKVVLDSDAGLF
	10	RDEVLPRIKKLGYNAVQJMAIQEHSYYAS
Starch debranching enzyme-related proteins	1	NDTHPTLAIPELMRJLIDVEGLGWDEAWDITEKTIAYTNHTVLPEALEKW
	2	SQHISTAGMEASGTSNMKFALNGCIJIGTLDGANVEIREEV
	3	DFPSYIDAQEKVDEAYKDKKKWTKMSILNTAGSGKFNSDRTIAZYAKDIW
	4	FADYLEIWPAKFQNKTNGITPRRWJRFCNPELSEIISKWJGSDDWVLNLD
	5	PDALFDIQIKRIHEYKRQLLNILGAVYRYKKLKGMSAEDRQKVTPPRVCI
	6	ANTLNCNHPVVQKLILDSLRHWVJDYHVDGFRFDLAPFLVR
	7	GZENFFLFGAEADZIAGLRKDRENGKFKPDPRFEEVKRFIRSGVFGTYDY
	8	AFATYCNAKRIVKFITDVGAVVNNDPDIGDLLKVVFIPDYNVSVAEVLIP

9	EGZYENLAVLHLRARQIRNFLVPLMVSZGVPMLYM
10	LYVVGPFPHWKRWAEWNGKYRDDVRKFIKGEDGIAG

Motifs were identified separately for each functional protein group using MEME. Motif numbers correspond to those shown in Figures 5 and 6. The listed sequences represent consensus motif patterns for each group. B, J, and Z indicate ambiguous amino acid positions predicted by MEME.

Supplementary Table S2. Primer sequences used for qRT-PCR analysis of representative starch metabolism-related genes.

Gene	Forward primer (5′–3′)	Reverse primer (5′–3′)	Amplicon size (bp)
OsAGPL1	AAAGAGGAGAACTGCTGGCT	CTGAGGGTGTACAGGAAGCA	140
OsAGPS2b	CGAAGGTTCTTGATGCTGAT	TGAAGGCGCAATAATAGAGG	126
OsSSI	TAACCCGTTTGCTGAGAAAG	GTCCTCTTGGGAGGGTCTAA	134
OsSSIIa	GACAATGGCATGATGCAGTA	ACCTGGATCACTTCAAGCTG	129
OsBEIIb	GGTGGTCTTGGACTCAGATG	GAACCTGCGTTGTCTATGCT	148
OsGBSSI	GAACAACTACCAGCCCAATG	GTCATCCTTCGATTTCATCG	146
OsActin	GTCCTCTTCCAGCCTTCCTT	ACATCGTTCTCAGTGGTGGT	129

Supplementary Table S3. Full genomic annotation and predicted physicochemical properties of starch metabolism-related genes identified in *indica* rice.

Gene name (*indica* sub.)	Locus No. (MBKbase)	Chr.	pI	Molecular weight (Da)	Instability index	Aliphatic index	GRAVY	Amino acid (aa)	Gene size (bp)	Chromosomal position
OsAGPL2	OsR498G0101598700.01	1	5.48	57574.64	38.17	82.28	-0.221	518	7,399	chr01:26494139..26501537 (+)
OsSSIVa	OsR498G0101909600.01	1	5.52	95554.02	42.22	88.65	-0.276	843	1,406	chr01:31085667..31087072 (+)
OsPHOH	OsR498G0102345000.01	1	6.81	94443.73	33.16	84.95	-0.318	841	6,260	chr01:37675218..37681477 (-)
OsBEIIb	OsR498G0203981600.01	2	5.65	54072.99	35.87	68.74	0.341	468	7,256	chr02:20577270..20584525 (-)
OsSSIIb	OsR498G0204761600.01	2	6.04	75559.33	40.92	79.99	-0.276	694	5,000	chr02:32996038..33001037 (-)
OsAGPL1	OsR498G0307002800.01	3	5.32	45721.97	31.56	89.71	0.184	411	4,829	chr03:33154139..33158967 (-)
OsSPHOL	OsR498G0307078200.01	3	5.35	97381.91	36.86	87.85	-0.356	860	7,073	chr03:34418945..34426017 (-)
OsBEIIa	OsR498G0408518200.01	4	5.05	94779.96	40.14	69.79	-0.482	841	9,226	chr04:20340269..20349494 (-)
OsSSIIIb	OsR498G0409312000.01	4	5.37	138264.65	49.52	72.41	-0.606	1,216	8,649	chr04:32184514..32193162 (-)
OsPUL	OsR498G0407601700.01	4	6.25	63113.97	32.78	83.18	-0.366	566	13,440	chr04:4060914..4063914 (+)
OsISA2	OsR498G0510777600.01	5	5.81	70959.76	38.83	87.79	0.008	643	2,441	chr05:20354426..20356866 (-)
OsSSIVb	OsR498G0511243300.01	5	6.03	104178.5	42.91	89.79	-0.402	915	8,305	chr05:27842555..27850859 (+)
OsAGPL3	OsR498G0511403400.01	5	6.34	57653.73	35.72	82.85	-0.212	519	6,288	chr05:30224259..30230546 (-)
OsGBSSI	OsR498G0611577100.01	6	8.16	66768.51	30.05	82.86	-0.197	611	5,035	chr06:1643113..1648147 (+)
OsSSI	OsR498G0611665000.01	6	5.89	70952.02	43.11	81.23	-0.185	641	7,750	chr06:2969884..2977633 (-)
OsSSIIa	OsR498G0611935700.01	6	5.28	88377.35	48.69	76.00	-0.412	810	4,981	chr06:6811239..6816219 (+)
OsBEI	OsR498G0613332800.01	6	5.93	70017.28	33.37	71.88	-0.464	605	8,448	chr06:32095419..32103866 (-)
OsGBSSII	OsR498G0714096400.01	7	6.38	67291.60	33.42	84.93	-0.100	608	8,062	chr07:13351148..13359209 (-)
OsGPT1	OsR498G0815333700.01	8	9.77	42018.51	45.95	102.84	0.482	387	4,215	chr08:4987410..4991624 (+)
OsSSIIIa	OsR498G0815345700.01	8	4.60	128008.71	45.66	78.12	-0.555	1,144	6,573	chr08:5210505..5217077 (+)
OsAGPS2a	OsR498G0815850400.01	8	6.58	56104.06	42.56	90.37	-0.159	514	7,907	chr08:16326352..16331258 (-)
OsAGPS2b	OsR498G0815850400.01	8	6.58	56104.06	42.56	90.37	-0.159	514	9,269	chr08:16326544..16332812 (-)
OsISA1	OsR498G0816481400.01	8	5.79	43135.43	35.51	64.66	-0.501	380	10,907	chr08:27297051..27304957 (-)
OsAGPS1	OsR498G0917015400.01	9	6.06	55003.83	37.41	89.48	-0.126	502	4,543	chr09:8097637..8102179 (-)
OsISA3	OsR498G0917656000.01	9	6.09	45713.51	46.74	77.17	-0.264	417	11,346	chr09:19330463..19341808 (-)
OsSSIIc	OsR498G1018786300.01	10	5.66	83337.30	45.07	85.03	-0.285	749	8,525	chr10:17488269..17496793 (+)

Supplementary Table S4. Full genomic annotation and predicted physicochemical properties of starch metabolism-related genes identified in *japonica* rice.

Gene name (*japonica* sub.)	Locus No. (RAP-DB)	Chr.	pI	Molecular weight (Da)	Instability index	Aliphatic index	GRAVY	Amino acid (aa)	Gene size (bp)	Chromosomal position
OsAGPL2	Os01g0633100	1	5.48	57574.64	38.17	82.28	-0.221	519	8,079	chr01:25353805..25361883 (+)
OsSSIVa	Os01g0720600	1	5.69	109889.96	44.36	84.88	-0.352	976	8,998	chr01:30032428..30041425 (-)
OsPHOH	Os01g0851700	1	6.81	94470.76	33.16	84.95	-0.321	842	6,158	chr01:36670321..36676478 (-)
OsBEIIb	Os02g0528200	2	5.69	92743.16	38.34	69.16	-0.416	826	11,338	chr02:19355790..19367127 (-)
OsSSIIb	Os02g0744700	2	6.05	74150.68	39.21	79.81	-0.307	678	4,919	chr02:31233292..31238210 (-)
OsAGPL1	Os03g0735000	3	7.01	55427.08	35.55	88.26	-0.129	512	5,204	chr03:30099369..30104572 (-)
OsSPHOL	Os03g0758100	3	5.61	109129.03	40.52	86.64	-0.321	979	7,131	chr03:31332033..31339163 (-)
OsBEIIa	Os04g0409200	4	4.72	37175.23	52.26	68.80	-0.59	344	3,250	chr04:20240211..20243460 (-)
OsSSIIIb	Os04g0624600	4	5.4	138204.60	49.70	72.32	-0.61	1,217	7,821	chr04:31751600..31759420 (-)
OsPUL	Os04g0164900	4	6.71	53309.41	32.18	87.00	-0.261	477	1,988	chr04:4405753..4418895 (+)
OsISA2	Os05g0393700	5	6.12	86530.18	41.41	84.35	-0.02	801	2,548	chr05:19155053..19157600 (-)
OsSSIVb	Os05g0533600	5	6.03	104178.5	42.91	89.79	-0.402	916	8,214	chr05:26485770..26493983 (+)
OsAGPL3	Os05g0580000	5	6.34	57653.73	35.72	82.85	-0.212	520	5,484	chr05:28871794..28877277 (-)
OsGBSSI	Os06g0133000	6	8.34	66476.22	30.23	83.14	-0.19	610	5,032	chr06:1765622..1770653 (+)
OsSSI	Os06g0160700	6	5.89	70952.02	43.11	81.23	-0.185	642	7,513	chr06:3079296..3086808 (-)
OsSSIIa	Os06g0229800	6	5.28	88375.39	49.64	76.12	-0.414	811	4,905	chr06:6748398..6753302 (+)
OsBEI	Os06g0726400	6	6.35	93236.34	36.15	68.59	-0.491	821	8,426	chr06:30897378..30905803 (-)
OsGBSSII	Os07g0412100	7	6.26	67354.78	32.67	86.69	-0.076	609	7,320	chr07:12916883..12924202 (-)
OsGPT1	Os08g0187800	8	9.77	42018.51	45.95	102.84	0.482	388	3,920	chr08:5139674..5143593 (+)
OsSSIIIa	Os08g0191433	8	4.99	174940.43	45.43	78.76	-0.474	1,554	11,172	chr08:5352105..5363276 (+)
OsAGPS2a	Os08g0345800	8	6.58	56104.06	42.56	90.37	-0.159	514	1,928	chr08:15666336..15671051(-)
OsAGPS2b	Os08g0345800	8	5.87	52950.43	37.92	92.65	-0.238	480	1,745	chr08:15666336..15672583 (-)
OsISA1	Os08g0520900	8	5.39	89660.96	40.92	69.20	-0.336	804	6,920	chr08:25893657..25900576 (-)
OsAGPS1	Os09g0298200	9	6.23	54844.69	37.86	89.44	-0.133	501	4,380	chr09:7245434..7249813 (-)
OsISA3	Os09g0469400	9	4.92	37699.45	39.44	78.17	-0.288	340	17,238	chr09:17851431..17868668 (-)
OsSSIIc	Os10g0437600	10	5.68	83312.29	45.99	84.91	-0.287	750	7,833	chr10:15673243..15681075 (+)

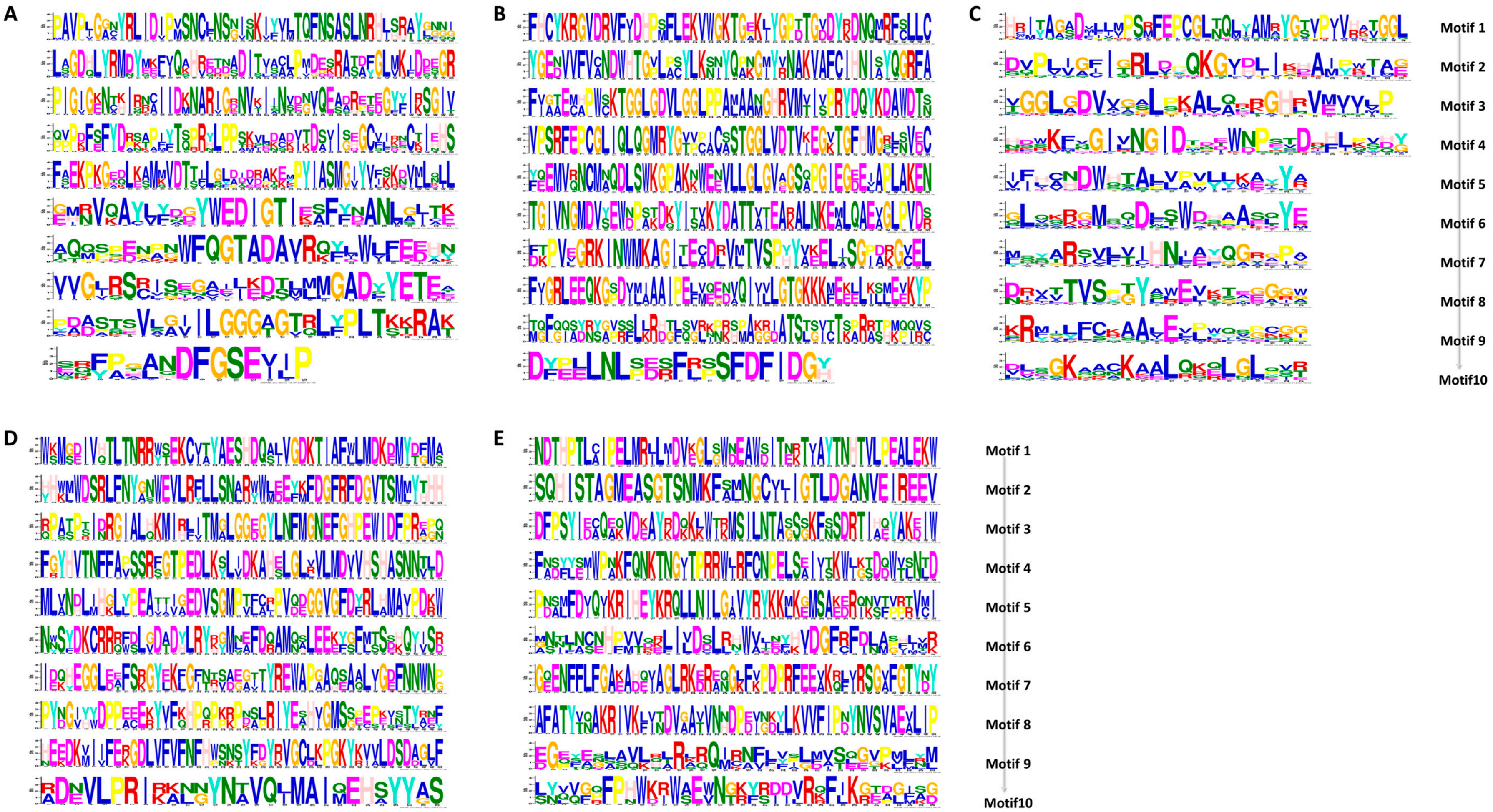

Supplementary Figure S1. Sequence logos of conserved motifs identified in starch metabolism-related proteins of *indica* and *japonica* rice. Motifs were predicted using MEME, and the sequence logos show the conserved amino acid patterns within each functional protein group. (A) ADP-glucose pyrophosphorylase-related proteins. (B) Granule-bound starch synthase-related proteins. (C) Soluble starch synthase-related proteins. (D) Starch branching enzyme-related proteins. (E) Starch debranching enzyme-related proteins.

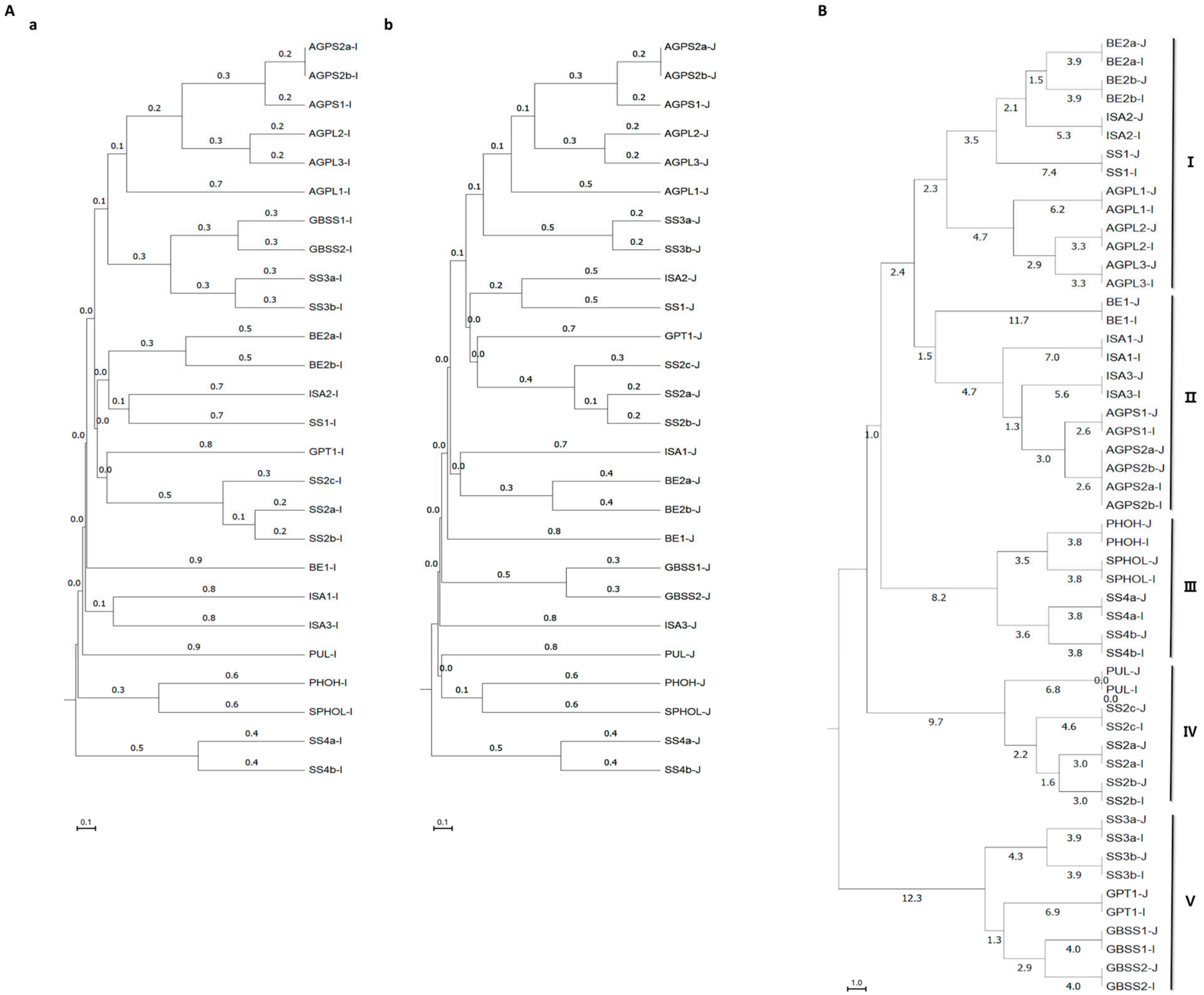

Supplementary Figure S2. Phylogenetic relationships of starch metabolism-related proteins identified in *indica* and *japonica* rice. Phylogenetic trees were constructed using the Neighbor-Joining method in MEGA11 with 1,000 bootstrap replicates. (A) Separate phylogenetic trees for *indica* and *japonica*. (B) Combined phylogenetic tree including proteins from both subspecies. The proteins were grouped into five major clades (I–V) corresponding broadly to the major functional categories of starch metabol ism-related proteins: clade I, branching and debranching enzymes; clade II, AGPase large subunits; clade III, AGPase small subunits and isoamylase-related prot eins; clade IV, starch phosphorylase family members and SSIV; and clade V, starch synthases together with GBSS and GPT1

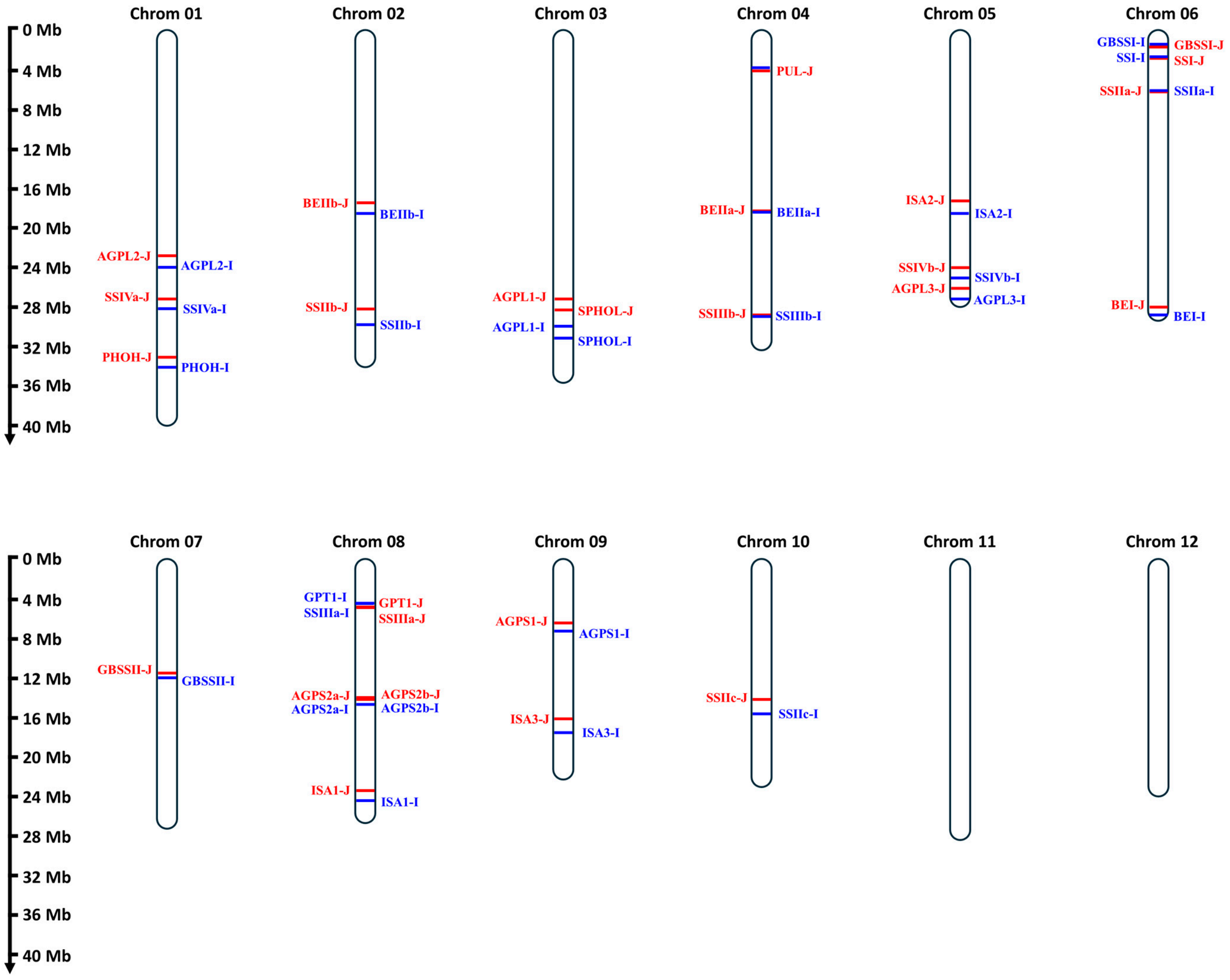

Supplementary Figure S3. Chromosomal distribution of starch metabolism-related genes in the two *Oryza sativa* subspecies, *indica* and *japonica*. Chromosome numbers are shown at the top, and the scale on the left indicates chromosome length (Mb). Gene names from *japonica* are shown in red, whereas those from *indica* are shown in blue.
